# Blood Examination in General Practice1Read by invitation before the Medical Society of the State of New York at its ninety-seventh annual meeting, held at Albany, January 27-29, 1903, being part of a symposium on hematology.

**Published:** 1903-06

**Authors:** Irving Phillips Lyon

**Affiliations:** Buffalo, N. Y.; 531 Franklin Street


### Blood Examination in General Practice.[1]

By IRVING PHILLIPS LYON, M. D., Buffalo, N. Y.

FIRST, I wish to express my high sense of appreciation of the honor done me by the officers of your ancient and honorable body by their invitation to me, a non-member, to address you. Your President suggested that the treatment of the subject assigned to me be practical and suited to the interest of the general practitioner rather than the pathologist or specialist in hematology, for which suggestion I was glad, as it permitted me to speak at all on this occasion. For I had no new discovery to announce to the pathologist, and though this would apply as well to the practitioner of medicine, yet I trusted to his greater leniency of judgment, as at least I should be a no worse offender in such respect than most others who read papers at general medical meetings. Though, therefore, I shall say nothing that has not been said before, I shall try to convince some skeptic amongst you, perchance, of the real utility of blood examination in the daily practice of his profession. For, in spite of all that has been spoken and written on this subject, it is true that the profession at large still have only slight familiarity with blood examination as a method of diagnosis.

I have drawn from my personal experience, in private and hospital practice, the following few cases which I have selected to narrate, with all possible brevity, for the purpose of giving concrete examples of the value of blood examination in determining diagnosis and thus rational treatment, whether medical or surgical. By this I do not intend to imply that blood exam-

1. Read by invitation before the Medical Society of the State of New York at its ninety-seventh annual meeting, held at Albany, January 27–29, 1903, being part of a symposium on hematology.

ination was the exclusive means of settling the diagnosis, but that it was an important, sometimes an essential factor among all other possible means of information in determining the diagnosis. For all available means of obtaining light on a case were invariably adopted and the blood was merely one, though an important one, of such means. And I would expressly state my conviction that a blood worker of any merit must be an even better clinician than hematologist, neglecting no sign or symptom and carefully correlating all the facts of a case before reaching a judgment. For one person must ultimately make the diagnosis and assume responsibility for it, and that person must combine the experience and matured judgment of both the clinician and the hematologist in order properly to correlate the whole evidence. The failure to recognise this principle has been the cause of much of the misunderstanding and discredit of blood reports and diagnoses based thereon.

I shall limit my remarks to citing only a few instructive cases, as examples, and to commenting thereon and perhaps on a few practical points of general interest in clinical blood examination.

*Central Pneumonia, Obscured by Pleural Effusion.*—This case occurred in my private practice in November, 1901, in a woman, Mrs. H., aged 75 years. The onset was sudden with chill and pain in the left side behind, following exposure to cold. The symptoms were those of pneumonia or acute pleurisy. The physical examination of the chest was almost negative and remained so until the end of the second day, when slight percussion dulness began to appear at the left base behind, without tubular breathing or râles. There was no expectoration. Next day friction sounds were heard over the relatively dull left base without other changes. On the fourth day flatness and positive physical signs of fluid were obtained and about 600 c.c. of clear serous fluid were aspirated. No tubular breathing or characteristic râles were then present. Was the case, then, one of simple acute pleurisy with effusion? No, it was a case of atypical pneumonia complicated by serous pleurisy, as made probable by the blood examination, which showed a high leucocytosis (35,000) even on the first day. The blood was examined daily and the leucocytosis persisted, with a relative increase of the neutrophiles, an iodine reaction in the leucocytes and a marked increase of fibrin deposit. I told the family, at first, my belief that the case was one of pneumonia, because the blood indicated it in spite of the negative physical signs. Not until the seventh day did tubular breathing and signs of pneumonic consolidation appear. By the next day, however, the process had extended and involved the entire left lung, which was solid and flat from top to bottom, with intense tubular breathing and râles over it everywhere. The patient died on the eleventh day.

Was not the blood examination of value in this case? It is in cases in which pneumonia is suspected and cannot be determined positively by other means that the blood offers assistance. By means of examination of the blood acute serous pleurisy and influenza can usually be differentiated from lobar pneumonia. Besides the usual leucocytosis I have found a positive and marked iodine reaction in the leucocytes in all cases of pneumonia, and I have come to regard this reaction of distinct value in the diagnosis of atypical cases.

*Pelvic Abscess.*—Mrs. D., seen with Dr. Herman E. Hayd at the German Deaconess Hospital, in September, 1899, had developed a septic temperature two weeks after the birth of her baby. This had continued for about one month in spite of repeated uterine curetting and douches. It seemed that the symptoms depended on a further cause than the slight uterine inflammation with its free drainage and active treatment. The blood examination showed 40,000 leucocytes, a relative neutrophilia, an increase of fibrin and a marked secondary anemia. The blood examination was distinctly in favor of abscess, and examination and operation, a few days later, located a pocket of pus between the uterus, bladder and omentum.

*Perinephritic Abscess.*—L.B., a boy of 8 years, I saw with Dr. Lorenzo Burrows about two years ago. He had been running an irregular temperature with an occasional slight chill for a few days, and had been seen in consultation by several physicians. Various possibilities of diagnosis had been considered, especially typhoid fever, malaria and tuberculosis. After a few days it was noticed that the boy lay in bed with his left thigh slightly drawn toward the abdomen and that deep pressure in the left flank caused him to wince. The urinalyses were negative. After about ten days of slight and indefinite symptoms, I was asked to examine the blood and found 33,000 leucocytes, the neutrophiles in excess, and a marked increase in fibrin. Upon inquiry as to the signs and symptoms of the case, I obtained the information already stated, and that appendicitis, pneumonia, empyema and pericarditis had been excluded, and further that nothing could be discovered except the slight tenderness in the left flank on deep palpation, the voluntary flexure of the left thigh and some discomfort to the patient when the thigh was actively or passively extended. In view of the blood picture, which was that of active inflammation, and of the localising symptoms described, I made a diagnosis of "deep-seated abscess in the left side, probably perinephritic." Two days later this diagnosis was confirmed by operation and by the discovery of about half a teaspoonful of pus behind the left kidney. The boy made a prompt recovery.

Who would deny the value of the blood examination in this case? Without it, days, perhaps weeks, might have passed before the diagnosis had become clear, and in the meantime serious damage to the kidney and other organs might have occurred.

*Appendicitis.*—Miss M. N., about 19 years of age, had been taken with nausea and vomiting while at dinner two nights before my examination. Fever, a coated tongue and diarrhea followed, with a little tenderness in the lower part of the abdomen, not sharply localised. Most of these symptoms subsided and the patient felt rather comfortable on the second day, a slight temperature, however, continuing. A very thorough examination of the appendix by Dr. D. W. Harrington, the physician in charge, and Dr. Matthew D. Mann, in consultation, at 4 o'clock on the afternoon of the second day, failed to reveal any positive signs of appendicitis, and it was thought that appendicitis probably did not exist. For caution's sake, however, a blood examination was considered advisable, which I made in the early evening of the same day, with the result that 20,000 leucocytes, a relative neutrophilia, an intracellular iodine reaction and an increase of fibrin were found. The blood indicated appendicitis, and, moreover, a well advanced stage of the disease. An immediate operation was done and an appendix in an early stage of gangrene was removed. The patient recovered promptly.

What might have happened had this appendix been left alone over night or longer? The blood examination possibly saved this young woman her life. This case illustrates the importance of blood examination as a routine procedure in doubtful cases of appendicitis. Physical examination alone failed to detect the disease, but blood examination was competent to show its presence. Such a single case as this is sufficient answer to the scoffing criticism of blood examination in appendicitis that we have heard so persistently from a certain quarter.

In another case of suspected appendicitis that I saw recently with Dr. DeLancey Rochester, there were 6,600 leucocytes, of normal differential distribution, a normal fibrin deposit and an absence of iodine reaction. In this case on the second day the symptoms were highly suggestive of appendicitis. The blood was flatly opposed to it, however, and the patient promptly recovered without any further trouble. Many similar cases might be cited, but one suffices for illustration.

*Intestinal Perforation in Typhoid Fever.*—I have had the opportunity recently of examining the blood in three cases of typhoid perforation in the Buffalo General Hospital, in the services of Drs. Charles G. Stockton and Charles Cary. In not one of these cases was a gross leucocytosis found, though a relative neutrophilia was present. In all three cases an intracellular iodine reaction was demonstrated. In spite, therefore, of the absence of leucocytosis, due to overwhelming septic infection by which the reactive power on the part of the blood is paralysed, the regular presence of iodophilia may be of material assistance in determining the diagnosis. As iodophilia may occasionally occur in typhoid independently of any septic complication, its presence with symptoms of perforation is not a certain sign of perforation, unless slips of blood previously taken showed its absence before the advent of signs suggesting perforation. This caution indicates the method of avoiding error.

*Acute Intestinal Obstruction.*—This case was seen last July with Dr. Charles P. Chapin. The patient, Mr. G., aged about 50 years, had suddenly developed symptoms of acute intestinal obstruction and finally typical fecal vomiting. The bowels had not moved for several days and had resisted repeated efforts to secure their evacuation. Exploratory laparotomy was considered and almost determined upon. The blood was examined and found entirely normal. This was against any serious pathological condition of the intestine and encouraged a waiting policy. A renewed effort to move the bowels by large and repeated enemata proved successful at last and was followed by a large number of passages of a surprising amount of fecal material. The patient was relieved of distress and made an immediate recovery. The blood examination was the chief factor in postponing and finally making unnecessary the proposed laparatomy.

*Trichinosis.*—Mr. B., a German, about 50 years old, I saw in consultation with Dr. E. R. Gould during last July. He had had a moderate temperature for a few days, felt rather sore in the calves of the legs, the eye-lids were slightly puffy and ecchymoses were noted in the conjunctivæ of both eyes. The blood showed 9,800 leucocytes, over 30 per cent. of which were eosinophiles. About two weeks before the patient had eaten Westphalian ham, it was subsequently ascertained. As no other cause of so marked an eosinophilia could be determined, after careful examination of the skin, stools, and the like, it was concluded in view of the general symptoms that the patient was suffering from a mild trichinous infection. Unfortunately, the opportunity was not afforded of confirming the diagnosis by examination of a bit of muscle from the calf.

*Malaria versus Cancer of the Kidney.*—In March, 1901, a woman about 60 years old was admitted to the Buffalo General Hospital, in the service of Dr. Roswell Park, for operation on what had been diagnosticated as cancer of the right kidney. A mass resembling the kidney could be felt in the right hypochondrium and the urine contained considerable blood. Moreover the patient had lost flesh, had grown weak and anemic and had had chills. Malaria had not been suspected. A glance at the fresh blood preparation showed half-grown tertian malarial organisms, and there was a marked anemia without leucocytosis. Upon inquiring it was found that the patient had come from Georgia about six months previously, had had malaria in Georgia, and had had chills on alternate days for about two weeks before entering the hospital, with bloody urine following the chills. The diagnosis looked clear—malaria, floating kidney and hematuria. Quinine was given, the chills and hematuria disappeared, the patient recovered strength and in a few days returned to her home, escaping an exploratory laparatomy.

*Malignant Disease of the Left Kidney versus Splenic Hyperplasia, Chronic Splenic Anemia, Etc.*—Every textbook of medicine lays down principles for the differentiation of splenic from renal enlargement, when a solid mass is encountered in the left upper abdominal quadrant. Such a differentiation is notoriously difficult in many cases, and any means of aid should be welcomed. I believe that such an aid is furnished by the blood. This has correctly guided me to a diagnosis in five cases of malignant tumor of the kidney, in four cases of chronic splenic anemia, in one case of splenic hyperplasia secondary to alcoholic portal cirrhosis, and in one case of splenic enlargement associated with and probably due to chronic passive congestion of cardiac origin, although this case, too, may perhaps be one of splenic anemia. I have met only one case in which the blood appeared to be a false guide and this case is still living and may perhaps, after all, accord with the rule.

The principle involved in the differentiation lies in the fact that malignant disease of the kidney is usually accompanied by a leucocytosis with relative neutrophilia, whereas on the contrary, simple splenic hyperplasia rarely shows leucocytosis and often distinct leucopenia. Malignant disease of the spleen would agree with malignant disease of the kidney in its blood picture and might cause confusion, except for the fact that primary malignant disease of the spleen is so rare as to be almost a pathological curiosity and may, therefore, be neglected in practice. Inflammatory mass of the kidney and large perinephritic abscess are more likely to confuse. As a rule, these conditions can be differentiated from cancer of the kidney by the history, symptoms, physical signs, urinalysis and by the presence of the blood picture of abscess, *i.e.*, marked leucocytosis, increased fibrin and iodophilia. Increased fibrin deposit and iodophilia together are not often found in the blood with malignant tumor of the kidney, though they may be if marked inflammatory degeneration involves the cancer of the kidney. Observing all these points, it is usually easy to distinguish abscess from malignant disease of the kidney. Leukemia could cause no trouble, as a mere glance at a stained specimen of the blood would suffice to distinguish it, except in rare cases.

The problem is thus narrowed down to deciding whether a solid mass in the left hypochondriac region is a malignant tumor of the kidney or a simple non-malignant hyperplasia of the spleen, and the blood examination is usually competent to decide. This guide must be applied with caution and judgment and in strict conjunction with a careful weighing of the evidence furnished by the history, symptoms and signs of the case. Thus applied it is of undoubted value, as evidenced by the following illustrative cases:

*Cancer of the Left Kidney.*—Case I.—Mrs. C., aged about 60 years, was referred to me by Dr. Matthew D. Mann, in May, 1899, with the statement that she had a solid mass in the region of the spleen. The blood showed 17,000 leucocytes, of which 89 per cent. were neutrophiles, a normal fibrin deposit and a moderate secondary anemia. I referred the patient back with the diagnosis, "cancer or inflammatory mass of the left kidney, probably cancer. The diagnosis of cancer of the kidney was fully confirmed, Dr. Mann subsequently stated.

Case II.—Mr. C., aged 39 years, was admitted to the Buffalo General Hospital, in the service of Dr. Roswell Park, in June, 1899, for operation on an "enlarged spleen of splenic anemia." The tumor occupied exactly the splenic region, projecting downward and forward below the costal margin on the left side. It was firm and solid, smooth, non-movable, and had an anterior edge that was somewhat narrow or sharp, resembling a spleen. This mass had been growing rapidly for five months at least. No blood had ever been detected in the urine. The patient complained chiefly of a soreness in the left side. The blood, taken on the morning of the operation showed as follows: red corpuscles, 3,804,000; hemoglobin, 51 per cent.; leucocytes, 14,700; neutrophiles, 86 per cent.; fibrin, normal; an occasional normoblast and moderate poikilocytosis. With such a blood picture, I followed the rule and made a diagnosis of cancer of the left kidney, as opposed to splenic enlargement. Dr. Park, however, in view of all the circumstances, determined to explore the abdominal cavity. The abdomen was opened, the spleen was found normal in size, the wound was closed, the patient was turned over, the kidney was cut down upon and found involved in a large cancerous growth.

This case is particularly interesting, as the mass felt exactly like a spleen, even after the patient was under complete anesthesia. Not a single point, except the blood examination, suggested kidney as opposed to spleen, and even the blood changes were rather slight. However, the operation showed that the blood, even in this case, was a truer guide than the physical signs. I might add that I have never encountered a case of malignant tumor of the kidney in which a hyperleucocytosis was constantly absent. However, such cases have been reported, though rarely, and their possibility must never be neglected.

*Splenic Anemia.*—On the other hand, the blood may be of distinct value in confirming the splenic nature of an abdominal mass in this region, as in the following case:

Mr. G. S., aged 59 years, I saw on February 21, 1899, in consultation with Dr. Carlton R. Jewett. A large mass with a sharp edge projected from the left costal border well down into the abdomen. For about one year, the patient had been growing weak and anemic. The blood showed a marked anemia and only 4,800 leucocytes. A second blood examination, three months later, showed a more advanced anemia and only 3,075 leucocytes. Two weeks after the second blood examination, the patient died and the necropsy showed a hyperplastic spleen of chronic splenic anemia.

*Simple Splenic Hypertrophy Secondary to Portal Cirrhosis.*—We occasionally see very large spleens in adults in various other conditions, such as the ague-cake spleen of chronic paludism, the enlarged spleen of portal congestion, that which rarely accompanies pernicious anemia, the marked splenic enlargement occasionally met with in portal cirrhosis, and that which is usually seen with Hanot's biliary cirrhosis. With the exception of the last, a rare condition, which is said to be associated usually with leucocytosis and which should offer no difficulty in diagnosis, these various conditions rarely present leucocytosis and indeed leucopenia is sometimes found, especially in pernicious anemia. The absence of leucocytosis is therefore useful by indicating the simple splenic nature of such enlargements as against malignant tumor of the kidney, as exemplified in the following case:

Mr. V. B., aged 40 years, consulted me in May, 1900, complaining of general weakness. He was a confirmed drunkard, and had thickened arteries, the edge of the liver could not be felt and a mass with a sharp edge was felt on the left side of the abdomen, reaching almost to the transverse umbilical line. The blood showed a fair degree of anemia of a secondary type, a leucopenia of 3,120, a normal differential leucocyte count and a normal fibrin deposit. A diagnosis was made of simple splenic hypertrophy, secondary, probably, to cirrhosis of the liver; possibly, to splenic anemia. The patient committed suicide on January 20, 1902, affording me the opportunity of examining his organs. The liver was a typical hob-nail liver of alcoholic cirrhosis and the spleen, smaller than when previously felt (730 grams), presented the picture of simple chronic hyperplasia, secondary to cirrhosis.

The general principle which we have been considering, it must be expressly stated, applies only to adults or at least to those past the first few years of life, for the blood of infants and young children varies widely from the standard of adult life, and in childhood a marked leucocytosis is commonly found in conditions in which adults show no leucocytosis. I have dwelt at some length upon the application of this principle for the differentiation of simple enlargement of the spleen from malignant disease of the kidney, because its practical value warrants, I think, its recognition. It must not be applied indiscriminately with blind faith, but as a guide, supplementary to other methods of diagnosis, it is distinctly useful.

Many additional examples of the assistance of blood examination in diagnosis might be cited, if time permitted, but the few already given serve the purpose of concrete illustrations, as well as many. These cases speak for themselves and justify all that has been claimed for blood examination as a valuable clinical method of diagnosis in general practice. If you will employ this method you will save lives and suffering. Is it worth the trouble?

531 Franklin Street.

